# Impact VR: Building Socioemotional Resilience in Youth with Conduct Disorder

**DOI:** 10.1007/s11121-025-01876-x

**Published:** 2026-01-15

**Authors:** Nicholas D. Thomson, Jessica J. James, Victoria Blondell, Robert Perera, Laura Hazlett, Scott Vrana

**Affiliations:** 1 Arche XR LLC, 2400 Old Brick Road, Glen Allen, VA 23060 USA; 2 Department of Surgery, Virginia Commonwealth University, Richmond, VA USA; 3 Department of Psychology, Virginia Commonwealth University, Richmond, VA USA; 4 Department of Biostatistics, Virginia Commonwealth University, Richmond, VA USA

**Keywords:** Conduct disorder, Emotion recognition, Socioemotional intervention, Prevention, Virtual reality

## Abstract

Adolescents with conduct disorder (CD) often exhibit deficits in emotion recognition, strained parent and peer relationships, and elevated social stress. This randomized controlled trial tested Impact VR, a brief, immersive socioemotional intervention, with the aim of building protective factors and improving socioemotional functioning among youth with CD. A total of 110 adolescents with CD (*M*_*age*_ = 13.79; 58% male) were randomized to either the Impact VR intervention or an active control. Outcomes were assessed at baseline, post-intervention, and 3-month follow-up, including emotion recognition accuracy (ER40 total and subscales: fear, sadness, anger, happy, neutral), self-reported peer relationships, parent relationships, and social stress. Mixed-effects models controlled for baseline scores. Impact VR produced significant improvements in ER40 total accuracy (*d* = 0.74, *p* < .001), with specific gains for fear (*d* = 0.54, *p* < .001), sadness (*d* = 0.75, p < .001), and anger (*d* = 0.50, *p* = .014). No group differences emerged for happy (*p* = .126) or neutral (*p* = .050). Impact VR participants also reported stronger peer relationships (*d* = 0.58, *p* = .002) and parental relationships (*d* = 0.54, *p* < .001), and reductions in social stress at the 3-month follow-up (*d* = 0.53, *p* < .001). Findings demonstrate that even brief, scalable interventions delivered through immersive virtual experiences can yield meaningful improvements in socioemotional functioning for adolescents with CD. Impact VR represents a promising, engaging, and developmentally sensitive addition to the prevention science toolbox.

Conduct disorder (CD) is a serious and chronic psychiatric condition that emerges during childhood or adolescence, characterized by a persistent pattern of aggression, rule-breaking, deceitfulness, and violations of social norms (American Psychiatric Association, 2022). Affecting an estimated 3–5% of youth globally, CD is associated with significant impairments in academic, emotional, and social functioning (Erskine et al., 2014). Particularly, youth with CD show critical deficits in their ability to identify and interpret the facial emotions of others (Kleine Deters et al., 2020; Martin‐Key et al., 2018). This deficit has major implications across the life course; longitudinal studies show that early-onset CD often persists into adulthood, elevating risk for criminal behavior, substance abuse, and personality disorders (De Brito et al., 2021; Fairchild et al., 2019). However, early intervention that targets the socio-cognitive mechanisms underlying interpersonal dysfunction may help alter these adverse life trajectories (Blair, 2013; Frick & Viding, 2009). While there are existing interventions to address the effects of CD, these interventions are often greatly stigmatized, inaccessible, or unappealing to CD populations and their caregivers (Koerting et al., 2013). New technological advancements present an avenue to address these challenges and effectively combat CD through innovative techniques and delivery methods that address the key mechanisms underlying this disorder.

A core area of dysfunction in CD is emotion recognition, particularly for negative emotions such as fear, sadness, and anger (Kleine Deters et al., 2020; Muñoz Centifanti et al., 2021; Thomson et al., 2025a). In normative development, the ability to identify facial emotions becomes increasingly accurate across childhood and adolescence as neural systems mature and social experiences broaden (Mondloch et al., 2003; Montirosso et al., 2010). Children first differentiate broad affective categories such as happy versus not happy, and later acquire the cognitive and perceptual skills needed to detect nuanced expressions of fear, anger, and sadness (Adolphs, 2006; Herba & Phillips, 2004). Among youth with CD, this developmental trajectory is often disrupted, leading to systematic misinterpretation or missed detection of emotional cues (Hunnikin et al., 2022; Muñoz Centifanti et al., 2021).

Within CD, there is considerable heterogeneity. Youth can be further diagnosed with the specifier limited prosocial emotions (LPE), also referred to as callous-unemotional (CU) traits, if they exhibit a persistent pattern of reduced guilt or remorse, diminished empathy, shallow or constricted affect, and limited concern about performance in important activities (APA, 2022). This distinction is clinically meaningful. Youth with LPE typically show pronounced deficits in recognizing distress cues such as fear and sadness, which map onto impairments in affective empathy, and the capacity to share and respond to others’ emotions (Dadds et al., 2018; Díaz-Vázquez et al., 2024). Youth without LPE often exhibit a different socioemotional profile characterized by difficulties in cognitive empathy, heightened threat sensitivity, greater irritability, and an increased likelihood of hostile attribution biases (Sebastian et al., 2021; Thomson et al., 2017). These patterns suggest that disruptions in both affective and cognitive empathy contribute to the broader emotion recognition difficulties observed across CD.

Although these subgroups differ in their socioemotional profiles, a unifying treatment target involves improving social-emotional skills that support accurate emotion decoding and more adaptive social-information processing. Enhancing emotion recognition can reduce affective empathy deficits common among youth with LPE, and can also support emotion regulation and reduce hostile interpretations among those without LPE (Thomson et al., 2017). As a result, emotion recognition represents a mechanistic point of intervention that may benefit the full range of youth with CD despite underlying heterogeneity in their developmental pathways.

Emotion recognition deficits are best understood within the broader framework of social information processing (SIP) theory, which describes how individuals encode, interpret, and respond to social cues (Crick & Dodge, 1994). The SIP model comprises six stages: encoding, interpretation, goal clarification, response generation, response evaluation, and behavioral enactment. Youth with CD often display biases at the earliest stages, demonstrating selective focus on hostile cues rather than benign cues, difficulty redirecting attention following hostile cues, and perhaps most notably misinterpreting neutral or ambiguous expressions as hostile (Dodge, 1993; Dodge & Pettit, 2003). These biases may also be guided and reinforced by social-cognitive mechanisms. Youth with CD may hold maladaptive schemas that can influence each step of SIP, thereby strengthening these biases (Li et al., 2024). Such “hostile attribution biases” can lead to retaliatory or coercive responses, especially in social situations perceived as threatening (Dodge et al., 1995). Deficits in emotion recognition are thought to directly impair cue encoding and interpretation (Li et al., 2023a, b), setting the stage for downstream errors in response selection and aggressive enactment. These distortions are not only predictive of aggression but also correlate with poor peer and family functioning, suggesting a cascading developmental impact (Lansford et al., 2006). Encouragingly, research indicates that emotion recognition skills are developmentally malleable and responsive to targeted intervention (Dadds et al., 2012; Hunnikin et al., 2022).

A growing body of research supports the effectiveness of emotion recognition training as a developmentally appropriate strategy for improving social functioning and reducing externalizing behaviors in youth with conduct problems (Thomson et al., 2025a). These targeted strategies are often integrated into cognitive-behavioral or social-emotional learning frameworks to address misperceptions or biases in decoding affective social cues. Research has shown that improving emotional understanding at age five predicts better peer relations and reduced behavioral problems at age nine (Izard et al., 2001). Among youth with conduct problems aged 12 to 16 years, emotion recognition training was shown to reduce hostile attribution bias and aggression (Li et al., 2023a, b). Research by Dadds et al. (2012) has shown that structured emotion training, including guided feedback, role-play, and repeated exposure, improved emotion recognition among youth (aged 6–16 years) with CU traits. Even a brief emotion recognition training program has been found to result in sustained improvement in emotion recognition among juvenile justice-involved youth aged 14 to 17 years, with the greatest and most sustained improvements seen among adolescents with elevated CU traits (Muñoz Centifanti et al., 2021). These gains are most robust when the training corrects specific biases, such as over-attributing anger to neutral faces (Li et al., 2023a, b). Recent research with children exhibiting disruptive behaviors (aged 7–11 years) and justice-involved adolescents (aged 12–18 years) suggests that emotion recognition skills are developmentally responsive, with even brief interventions yielding improvements in emotional decoding and reductions in externalizing behavior (Hubble et al., 2015; Hunnikin et al., 2022; Wells et al., 2021). These findings demonstrate that targeted, brief interventions can recalibrate social-cognitive mechanisms underlying antisocial behavior and promote developmental catch-up.

While traditional evidence-based interventions for CD, such as parent management training, multisystemic therapy, and cognitive-behavioral therapy, have demonstrated effectiveness in reducing disruptive behaviors (Thomson et al., 2017), they primarily emphasize behavioral management and improvements in the parent–child relationship (Fairchild et al., 2019). These approaches often succeed in addressing overt conduct problems but tend to overlook the emotion-processing deficits and social-cognitive distortions that underlie aggression and antisocial behavior. More recent adaptations, such as Parent–Child Interaction Therapy for callous–unemotional traits (PCIT-CU; Fleming et al., 2022; Kimonis et al., 2019), represent an important step toward targeting these mechanisms by integrating emotional understanding and empathy training into parent-based treatment. However, even with these advances, engagement and treatment retention remain modest, with clinician-led programs reporting treatment dropout rates of 23%–26% (Fleming et al., 2022; Kimonis et al., 2019). Recent research investigated how adolescents with CD (aged 10–17 years) and their caregivers (*N* = 40) perceived existing mental health treatments (Thomson et al., 2025a). Youth highlighted several persistent barriers to conventional treatment, including feelings of stigma, disengagement, and a lack of cultural relevance. Nearly 90% of youth reported feeling ashamed or embarrassed when receiving traditional services, particularly when treatment occurred in visible or institutional settings such as schools or probation offices. The majority expressed skepticism about therapists' ability to understand their daily experiences, and many described feeling judged. Parents echoed this frustration, citing long waitlists, lack of accessible school-based options, and minimal behavioral improvements as key limitations of existing services (Thomson et al., 2025a). Notably, all of the 40 parents in the study reported that their child had either avoided or actively fled from treatment at some point (Thomson et al., 2025a). These combined barriers of stigma, restricted access, low motivation, poor retention, and system constraints create a treatment landscape that fails to meet the unique developmental and motivational needs of youth with CD. These challenges, combined with the extensive costs and limited scalability of clinician-delivered programming, highlight the need for next generation interventions that preserve mechanistic precision while improving accessibility, engagement, and sustainability. One promising solution lies in leveraging technology to meet youth where they are, using interactive and immersive platforms capable of sustaining motivation, reducing stigma, standardizing delivery quality, and enabling repeated, experiential emotion-recognition practice in realistic social contexts, while being deployable across settings without the need for extensive clinical staff or infrastructure (Blondell & Thomson, 2025; Kevorkian & Thomson, 2024).

Given these persistent barriers to engagement and scalability, efforts have turned toward digital and game-based interventions as alternative delivery methods for youth mental health care. A large scoping review of digital game interventions for youth mental health identified 49 studies that tested 32 distinct games and organized the findings within a stepped-care framework (Ferrari et al., 2022). The study found that most of the quantitative studies reported significant improvement in at least one targeted mental health outcome. The review emphasized that games are most effective when they incorporate user-centered design, therapeutic modeling, and gamified engagement features such as feedback and reward systems. However, most identified interventions focused on internalizing disorders, particularly depression and anxiety, and comparatively few targeted externalizing problems or aggression (Ferrari et al., 2022). A complementary meta-review by Vié et al. (2024) reached similar conclusions, noting that effective digital games for young people tend to integrate social-emotional learning elements, adaptive feedback, and skill rehearsal components that enhance motivation and self-efficacy. Together, these findings indicate that while the field of digital mental health gaming has advanced substantially, gaps remain in addressing conduct problems and socioemotional deficits central to disruptive behavior disorders. These gaps have created a clear opportunity for immersive technologies, such as virtual reality (VR), to build upon existing digital interventions by offering immersive, emotionally engaging environments where youth can actively practice socioemotional skills with real time feedback.

VR is a promising approach to addressing social-emotional development, mental health, and aggressive behavior (Kevorkian & Thomson, 2024). VR offers realistic experiences and control over environmental factors, which can enhance learning outcomes and support the generalization of skills to real-world contexts (Parsons, 2016). By facilitating first-hand experiences, VR simulations empower users to develop skills tailored to their specific needs through repeated practice (Sethi & Jain, 2024). Meta-analytic evidence has demonstrated a positive relationship between VR interventions and improvements in emotion recognition (Farashi et al., 2024) and social-emotional learning (Zhang et al., 2024). Among incarcerated adults, prior research has found that VR interventions can reduce self-reported and observed aggression (Woicik et al., 2023). Although these previous studies have included populations with autism, social anxiety, social problems, or typically developing youth, interventions for youth with CD are notably absent.

In response to the needs of youth with CD and the increase in technological capabilities, Impact VR was developed to address these systemic and engagement barriers through brief, interactive modules that teach social-emotional skills (i.e., emotion recognition and interpersonal skills) in a developmentally appropriate, immersive format. Grounded in SIP theory and informed by evidence linking CD to deficits in emotional processing, the program places youth in emotionally charged interpersonal scenarios where they are tasked with identifying affective cues and selecting prosocial responses. Gamified elements and immediate feedback enhance engagement, while psychoeducation is woven into interactive decision-making tasks. Building on the findings from Ferrari et al. (2022) and Vié et al. (2024), Impact VR was designed to integrate the features identified as most effective in prior digital-game research, including user-centered design, adaptive feedback, and social-emotional skill rehearsal, while extending these principles to the externalizing domain through immersive, interactive scenarios tailored for adolescents with CD. Development followed a participatory design model involving youth with lived experience, caregivers, educators, and mental-health providers to ensure relevance and cultural sensitivity. The resulting program received positive feedback from key stakeholders. The study involved 20 youth with CD (10–17 years) and their caregivers, 20 mental health professionals, and 20 educators (Thomson et al., 2025a). Across all groups, Impact VR received approval ratings exceeding 95% for acceptability, appropriateness, and feasibility. Youth participants overwhelmingly endorsed Impact VR as culturally sensitive, enjoyable, and a good use of their time, with 100% reporting that they prefer it to traditional counseling or case management, and 90–100% believing it would improve their relationships with parents, teachers, and peers. Caregivers and professionals echoed these sentiments, rating the intervention as resource-efficient and easily deployable across home, school, and clinic environments. Importantly, participants noted that the self-guided and private nature of VR delivery may help reduce the stigma often associated with traditional mental health services, particularly for youth reluctant to engage in therapy. These findings underscore Impact VR’s potential to overcome traditional barriers to treatment engagement and highlight its promise as a scalable, low-resource intervention that is not only feasible and well-received but also perceived as meaningful and relevant by both youth and key community stakeholders (Thomson et al., 2025a).

Drawing from the same sample as the present study, the first RCT study of Impact VR involving 110 youth with CD (ages 10–17 years) demonstrated that Impact VR reduced self- and caregiver-reported CU traits, conduct problems, and reactive aggression (Thomson et al., 2025b), and these improvements were sustained by the 3-month follow-up. This study provides preliminary evidence that Impact VR can address critical risk factors for CD and antisocial behavior. Building on this, the present study investigates whether Impact VR can also enhance protective factors that may buffer against these risks, specifically socioemotional skills such as emotion recognition, the quality of parent–child and peer relationships, and the ability to manage social stress. By investigating these positive youth developmental pathways, this study aims to advance understanding of how technology-based, mechanistically informed interventions can not only reduce risk but also promote resilience in youth with CD.

## The Present Study

This randomized controlled trial (RCT) tested the efficacy of Impact VR in strengthening protective factors among adolescents with CD. Primary outcomes included accuracy on a standardized emotion recognition task, youth-reported quality of parent and peer relationships, and levels of social stress. Previous research has demonstrated that Impact VR is feasible and acceptable for this population and reduces risk factors for CD; the current trial extends that evidence by evaluating its impact on socioemotional outcomes that function as buffers against risk trajectories. By placing youth in immersive, emotionally salient scenarios that replicate real-world interpersonal challenges, the study examined whether a VR-based approach can promote durable skill development in a group whose developmental pathways are often compromised by socioemotional deficits.

## Methods

### Participants

The study enrolled 110 adolescents (55 in Impact VR, 55 in the control group) recruited from a large urban healthcare system in Virginia. All participants were between 10 and 17 years old (*M* = 13.79, *SD* = 2.33). The sample was 58% male and 42% female. With respect to race and ethnicity, 58% identified as Black, 33% as White, and 9% as another racial background (e.g., Asian, Native American, Pacific Islander). Comorbid psychiatric conditions were common: 33% had ADHD, 10% had autism spectrum disorder, 13% were diagnosed with an anxiety disorder, 10% with post-traumatic stress disorder, and 16% with major depressive disorder. Table 1 presents demographic characteristics along with baseline scores for all study variables. Eligibility criteria required that participants speak English, be within the target age range, and have a current diagnosis of conduct disorder made by a licensed clinician.

**Table 1:** Descriptives for full sample and intervention groups

	Full Sample (*N* = 110)	Impact VR (*n* = 55)	Control Group (*n* = 55)
Mean age (*SD*)	13.79 (2.33)	13.91 (2.27)	13.69 (2.43)
Sex			
Male	64 (58%)	33 (60%)	31 (56%)
Female	46 (42%)	22 (40%)	24 (44%)
Ethnicity*			
Black	64 (58%)	33 (60%)	31 (56%)
White	37 (33%)	18 (32%)	19 (34%)
Other	10 (9%)	4 (8%)	5 (10%)
Comorbid Diagnoses			
ADHD	37 (33%)	20 (36%)	17 (30%)
Autism	11 (10%)	7 (12%)	4 (8%)
Anxiety Disorder	14 (13%)	8 (14%)	7 (12%)
Post-Traumatic Stress Disorder	11 (10%)	4 (8%)	7 (12%)
Major Depressive Disorder	18 (16%)	10 (18%)	8 (14%)
Mean Baseline Scores (*SD*)			
ER 40 Total Correct	29.75 (7.05)	29.75 (6.66)	29.74 (7.52)
ER 40 Happy Correct	7.73 (0.70)	7.78 (0.42)	7.69 (0.89)
ER Faces Sad Correct	5.34 (2.24)	5.20 (2.29)	5.46 (2.23)
ER Faces Angry Correct	4.53 (1.71)	4.53 (1.76)	4.54 (1.69)
ER Faces Fear Correct	5.92 (2.39)	6.06 (2.32)	5.80 (2.48)
ER Faces Neutral Correct	6.26 (2.24)	6.18 (2.30)	6.30 (2.20)
Peer Relationship T-score	53.67 (10.77)	52.71 (10.85)	54.24 (10.73)
Parental Relationship T-score	38.78 (14.00)	38.43 (15.11)	39.36 (13.09)
Social Stress T- Score	55.90 (12.57)	54.50 (11.75)	56.69 (13.03)

*Other = Asian, Native American, Pacific Islander

### Procedure

Youth were eligible if they were 10–17 years of age, English-speaking, and had a current diagnosis of CD, confirmed by a licensed mental health provider. Recruitment occurred through a large urban healthcare network in Virginia. Families who expressed interest after initial phone and email contact were scheduled for a baseline laboratory visit. At baseline, caregivers provided written consent and youth gave assent, each in private to ensure confidentiality. Participants were informed before enrollment that random assignment would be used. Youth then completed a battery of self-report questionnaires in a quiet setting (approximately 45 min). Randomization to the intervention or control condition was carried out using block randomization with variable block sizes (4, 6, or 8) to preserve balance across groups (Efird, 2011). The research coordinator responsible for baseline assessments remained unaware of the assignment until after those assessments were complete.

**Fig. 1:**
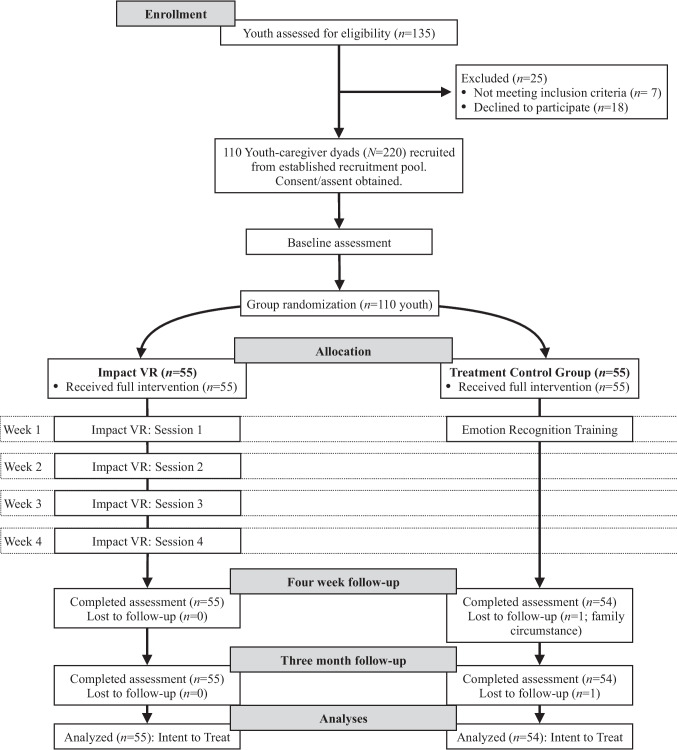
CONSORT diagram of Study (Thomson et al., 2025b)

Youth in the control condition participated in a one-time training session on emotion recognition (see Control Condition). Those assigned to Impact VR began the first of four weekly VR sessions in the lab, with the subsequent three sessions completed at home. Follow-up assessments were conducted at two time points: 4 weeks post-randomization (called post-intervention) and 3 months post-randomization. Both youth and caregivers contributed data at each follow-up, and each respondent received $75 compensation per assessment. The trial protocol was implemented without modification, approved by the university’s Institutional Review Board, and preregistered on ClinicalTrials.gov (NCT06301516) prior to participant enrollment in accordance with transparency and replication guidelines. This randomized controlled trial adheres to CONSORT 2010 reporting guidelines. Participant flow, enrollment, allocation, and retention are summarized in the CONSORT diagram (see Fig. 1).

### Interventions

#### Treatment Control

To provide a meaningful comparison, participants assigned to the control group completed an active emotion recognition intervention rather than a passive condition. A one-time session was selected because prior research has consistently shown that single-session emotion recognition training can produce measurable improvements in affective decoding. Brief, one-time interventions have been found to improve fear recognition in youth with conduct problems and CU traits (Dadds et al., 2006; Muñoz Centifanti et al., 2021), and similar single-session formats have produced gains in adults (Reed et al., 2023), including clinical samples such as adults with depression (Preis et al., 2025). These studies demonstrate that emotion recognition skills are malleable within a single session, providing a strong empirical basis for using a brief, standardized comparator that is credible, mechanism-relevant, and time-limited.

The control activity consisted of a single, approximately 20-min session delivered in a slideshow format by a study coordinator. Content was divided into two instructional components. The first emphasized where to direct attention when interpreting facial emotions, highlighting that the most informative cues are typically in the eyes and mouth. Illustrated examples were provided for four target emotional faces, including happiness, sadness, anger, and fear, as well as the neutral facial expression. Each emotion was paired with visual markers pointing to the facial regions most diagnostic of that expression. The second component explained how to recognize each of the four emotions by describing the facial muscle movements and configurations that typically signal them. For instance, fear was described as characterized by raised eyebrows, widened eyes, and an open mouth, while sadness was depicted through sloped brows, downcast eyes, and a frowning mouth. The session concluded with a brief summary encouraging participants to apply these strategies when observing others. To reinforce learning, the coordinator asked participants to indicate which features they would focus on to identify each emotion. Immediate corrective feedback was provided as needed. This format closely followed prior research protocols while ensuring youth engagement throughout the session.

### Impact VR

Impact VR (Arche XR, 2024) is a brief, immersive virtual reality program designed to build social-emotional skills in adolescents with conduct disorder. The intervention targets three core domains; emotion recognition, regulation, and prosocial responding, by placing participants in interactive, emotionally charged situations that mirror real-life social challenges. Using 360-degree VR technology, youth navigate dynamic scenarios that require identifying facial expressions, recognizing triggers, and selecting adaptive strategies for managing conflict. The platform integrates principles from cognitive-behavioral and dialectical behavior therapies but delivers them through a gamified and experiential format that emphasizes active learning and engagement.

Tasks are adaptive with difficulty levels adjusting in real time to sustain motivation and reduce frustration. Participants practice decoding emotions across varied conditions, including when cues are partially hidden (e.g., behind masks or sunglasses). In addition, narrative-driven problem-solving modules allow youth to test prosocial options in peer and group contexts, strengthening generalization of skills. Consistent with prior research on VR and emotional memory (Mancuso et al., 2023), the immersive quality of Impact VR is intended to increase ecological validity and promote consolidation of learned skills. The program is self-guided but can also be facilitated by a clinician or educator, and its design makes it deployable across schools, clinics, and community environments.

The intervention is delivered in four sessions of approximately 25 min each, with content becoming progressively more complex:

- *Session 1: Foundations of Emotion Recognition*.Youth are introduced to basic emotional expressions (happiness, sadness, anger, fear, neutral) and practice identifying them using facial features such as the eyes and mouth. Training includes mirroring, static/dynamic recognition tasks, and immediate feedback.
- *Session 2: Emotional Awareness and Adaptability*.Cues are made more challenging by obscuring features, and youth begin practicing regulation strategies. Gamified activities reinforce identification of emotional triggers and appropriate responses under time pressure.
- *Session 3: Emotional Skills in Social Settings.*Youth apply recognition and regulation skills within a collaborative narrative, navigating peer interactions and resolving conflicts. Role-play scenarios highlight empathy, perspective-taking, and constructive problem solving.
- *Session 4: Mastery in Complex Contexts*.Youth manage overlapping emotional signals and higher-intensity interpersonal challenges, integrating all prior skills. Advanced strategies for de-escalation and conflict resolution are rehearsed, culminating in a final interactive challenge requiring real-time decision making.

**Fig. 2:**
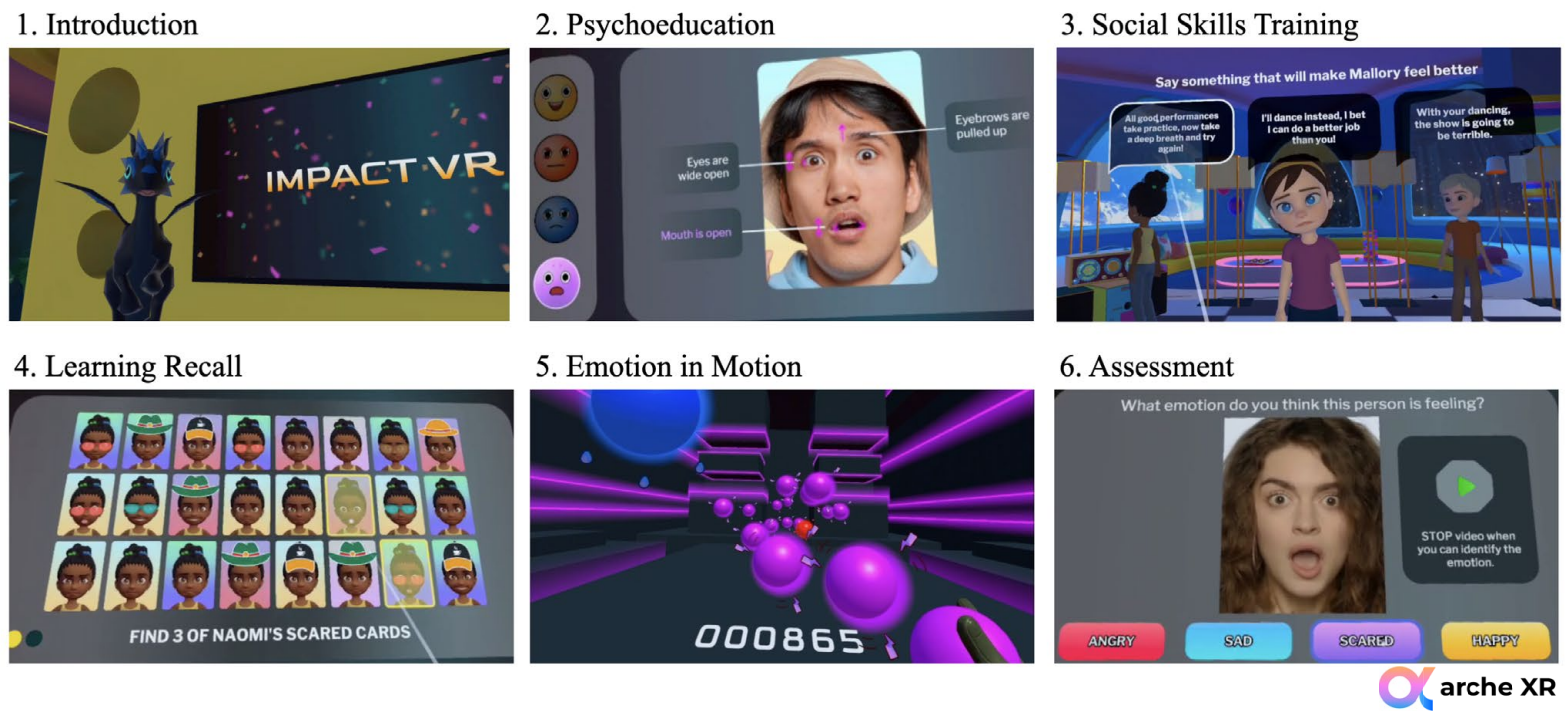
Session modules for Impact VR (Thomson et al., 2025b)

Each session follows a consistent sequence: a virtual companion introduces the day’s goals and reviews prior skills; participants then complete interactive learning tasks with feedback, followed by narrative-based practice scenarios. Sessions conclude with two reinforcement activities: a recall task that consolidates key concepts and the “Emotion in Motion” game, a fast-paced, music-synchronized task requiring participants to match emotional cues to targets for points. This structure is intended to maximize both skill acquisition and user engagement (see Fig. 2). For further details on design and participatory development, see Thomson et al. (2025a). No technical issues occurred across sessions with all participants completing each session without interruption.

### Measures

#### Emotion Recognition Task

The Penn Emotion Recognition Task (ER40; Kohler et al., 2003) is a standardized facial emotion recognition task consisting of 40 color photographs of adult faces displaying four basic emotions (happiness, sadness, anger, and fear), as well as neutral expressions. Each emotion category includes four high-intensity and four low-intensity expressions, and stimuli are balanced for the poser’s sex, age, and ethnicity. Participants view each image individually and are asked to select the emotion label that best describes the facial expression from a fixed set of five options. Responses are untimed, and no corrective feedback is provided. The primary outcome measure is the total number of correct responses (range: 0–40), with higher scores indicating greater accuracy in facial emotion recognition.

### Relationships and Social Stress

Youth perceptions of their social experiences and relational functioning were assessed using the Interpersonal (peer) Relations, Relations with Parents, and Social Stress subscales from the BASC-3 Youth Rating Scales (Reynolds & Kamphaus, 2015). Each subscale includes items rated on a 4-point Likert scale from 0 (Never) to 3 (Almost Always), with T-scores computed to facilitate interpretation. The peer relations subscale evaluates the youth’s ability to establish and maintain friendships with peers, as well as the quality of peer relationships (e.g., “Has trouble making friends,” “Gets along well with others”). Higher scores reflect stronger interpersonal functioning and more positive peer interactions. This subscale demonstrated good internal consistency in the current sample (α = .86). The Relations with Parents subscale assesses perceived closeness, trust, and communication between the youth and their caregivers (e.g., “I feel close to my parents,” “My parents listen to me”). Higher scores indicate more supportive and positive parent–child relationships. This subscale demonstrated acceptable internal consistency (α = .81). The Social Stress subscale captures the youth’s experience of anxiety or discomfort in social settings, including concerns about peer judgment and rejection (e.g., “Worries about being left out,” “Feels uncomfortable around others”). Higher scores represent greater social stress. This subscale demonstrated strong internal consistency (α = .89).

### Power Analysis

The study was powered based on the planned sample size of 110 adolescents. Calculations assumed an attrition rate of approximately 15% per condition and a within-subject correlation of 0.50 across repeated assessments. Under these assumptions, the design provided 80% power to detect a medium effect size (*f* = 0.25) for the main effect of group at α = .05, assuming no group × time interaction. When testing interactions, the study retained 80% power to detect a standardized mean difference of *d* = 0.48 at a Bonferroni-adjusted α = .025 for comparisons at the two follow-up points. Both thresholds are consistent with conventional definitions of medium effects.

### Data Analytic Plan

Descriptive statistics were calculated for participant characteristics and study outcomes, with means and standard deviations reported for continuous variables and counts with proportions for categorical variables. Summaries were provided for the full sample as well as by treatment group. Prior to model fitting, assumptions were evaluated visually using scatterplots and Q-Q plots. To evaluate intervention effects, linear mixed-effects models were estimated separately for each outcome. Each model included fixed effects for group (Impact VR vs. control), time (post-intervention and 3-month follow-up), and the group × time interaction, along with the baseline value of the outcome as a covariate. A random intercept was specified to account for repeated assessments within individuals. Our testing approach first examined the group × time interaction at α = .05. When this interaction was significant, we probed group differences at each time point using a Bonferroni-adjusted threshold (α = .025). If the interaction was non-significant, the main effect of group was interpreted as the estimated difference between intervention and control conditions averaged across time points. Mixed-effects models offer the advantage of handling both missing completely at random and missing at random data through maximum likelihood estimation; therefore, all participants with at least one assessment were retained in the analyses. Standardized effect sizes were also computed, expressed as Cohen’s d using the baseline standard deviation for each measure to facilitate interpretation (small = 0.20, medium = 0.50, large = 0.80; Cohen, 2013). All analyses were conducted in R (R Core Team, 2025) using the lme4 package.

## Results^1^

### Emotion Recognition

**Fig. 3:**
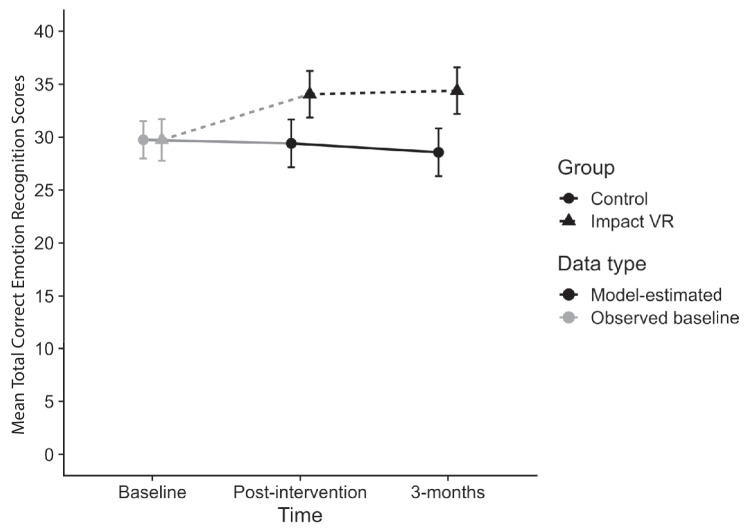
Total ER-40 correct scores at baseline, post-intervention, and 3 month follow-up by group. Post intervention and 3 month follow-up values reflect estimated marginal means adjusted for baseline scores. Baseline values reflect mean scores. Horizontal offset applied for visual clarity only

The mixed model for ER40 total correct scores found no significant group by time interaction (*p* = .373), indicating that group differences did not vary across follow-up. There was a significant main effect of group (*p* < .001), with youth in the Impact VR group scoring an average of 5.23 points higher than controls. No significant main effect of time was observed, indicating that differences between groups were consistent over the 3-month follow-up. The standardized mean difference was *d* = .74, reflecting a moderate-large effect (Fig. 3).

Analyses of specific emotion categories showed similar results. Youth in the Impact VR group identified more fear (difference = 1.30, *p* < .001, *d* = .54), sad (difference = 1.52, *p* < .001, *d* = .75), and angry faces (difference = 0.86, *p* = .014, *d* = .50) than the control group. These group differences were present immediately after the intervention and remained stable through the 3-month follow-up. For happy faces, there was no significant group difference (*p* = .126). For neutral faces, the group effect approached significance (*p* = .050) (Tables 2 and 3).

**Table 2:** ANOVA tables from the results of the mixed models

	Mean Square	Num DF	Den DF	*F*	*p*
ER-40 Total Correct Responses					
Group	289.063	1	97.07	12.97	0.0005
Time	3.456	1	97.41	0.16	0.6946
Group X Time	17.847	1	97.41	0.80	0.3731
Baseline Total Correct Response	14.432	1	96.84	0.65	0.4230
ER-40 Happy Correct Responses					
Group	1.40	1	102.00	2.38	0.1263
Time	1.15	1	103.00	1.96	0.1650
Group X Time	1.80	1	103.00	3.06	0.0834
Baseline Happy Correct Response	0.42	1	102.00	0.72	0.3996
ER-40 Fear Correct Responses					
Group	28.35	1	96.11	16.44	0.0001
Time	5.10	1	96.58	2.96	0.0888
Group X Time	0.33	1	96.58	0.19	0.6639
Baseline Fear Correct Response	0.07	1	95.84	0.04	0.8365
ER-40 Sad Correct Responses					
Group	1.30	1	102.00	19.83	< 0.0001
Time	1.49	1	103.00	0.71	0.4002
Group X Time	2.63	1	103.00	1.26	0.2638
Baseline Sad Correct Response	1.56	1	102.00	0.75	0.3881
ER-40 Angry Correct Responses					
Group	16.02	1	102.00	6.32	0.0135
Time	0.08	1	103.00	0.03	0.8607
Group X Time	5.15	1	103.00	2.03	0.1574
Baseline Angry Correct Response	0.04	1	102.00	0.02	0.8979
ER-40 Neutral Correct Responses					
Group	9.80	1	104.00	3.92	0.0503
Time	0.67	1	105.00	0.27	0.6045
Group X Time	0.00	1	105.00	0.00	0.9807
Baseline Neutral Correct Response	1.29	1	104.00	0.52	0.4744

**Table 3:** Estimated means from mixed models for ER40

Group	Mean	s.e	Lower	Upper
Total Correct Marginal Means				
Control	29.00	1.04	26.90	31.1
Impact VR	34.20	1.01	32.20	36.2
	Mean	s.e	*t*	*p*
Difference	− 5.23	1.45	− 3.60	0.0005
Correct Fear Marginal Means				
Control	5.98	0.23	5.52	6.43
Impact VR	7.27	0.22	6.83	7.72
	Mean	*s.e*	*t*	*p*
Difference	− 1.30	0.32	− 4.05	0.0001
Correct Sad Marginal Means				
Control	5.22	0.24	4.74	5.71
Impact VR	6.74	0.24	6.27	7.21
	Mean	s.e	t	p
Difference	− 1.52	0.34	− 4.45	< 0.0001
Correct Angry Marginal Means				
Control	4.71	0.25	4.22	5.19
Impact VR	5.56	0.24	5.09	6.04
	Mean	s.e	*t*	*p*
Difference	− 0.86	0.34	− 2.51	0.0135
Correct Neutral Marginal Means				
Control	6.06	0.27	5.53	6.60
Impact VR	6.80	0.26	6.29	7.31
	Mean	s.e	*t*	*p*
Difference	− 0.74	0.37	1.98	0.0503

### Peer and Parental Relationships

For interpersonal relationship T-scores, there was no significant group by time interaction (*p* = .904). A significant group effect was observed (*p* = .002), with Impact VR participants reporting scores that averaged 6.25 points higher than control group participants (*d* = .58). This difference was stable across the post-intervention and 3-month follow-up assessments.

**Fig. 4:**
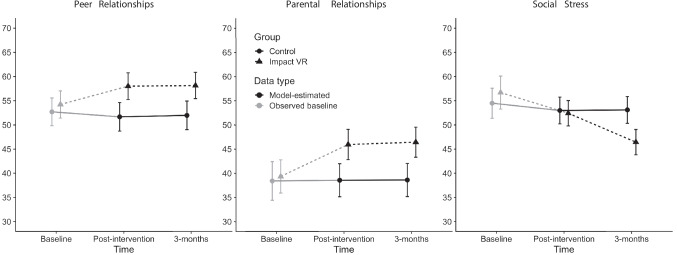
Self-reported peer relationships, parental relationships, and social stress at baseline, post intervention, and 3-month follow-up by group. Post-intervention and 3-month follow-up values reflect estimated marginal means adjusted for baseline scores. Baseline values reflect mean scores. Horizontal offset applied for visual clarity only

The same pattern was found for parental relationship T-scores. The group by time interaction was non-significant (*p* = .803), while the main effect of group was significant (*p* < .001). Youth in the Impact VR group reported parental relationship scores that averaged 7.61 points higher than controls (*d* = .54). No time effects were observed, indicating that group differences persisted over follow-up (Fig. 4).

### Social Stress

For social stress, there was a significant group by time interaction (*p* = .008). At the post-intervention time point, groups did not differ (*p* = .771, *d* = .04). By the 3-month follow-up, however, youth in the Impact VR group reported social stress scores that were 6.66 points lower than youth in the control group (*p* < .001, *d* = .53). This indicates that reductions in social stress emerged over time following the intervention (Tables 4 and 5).

**Table 4:** ANOVA tables from the results of the mixed models for Peer Relationships, Parental Relationships, and Social Stress

	Mean Square	Num DF	Den DF	*F*	*p*
Peer Relationships T-score					
Group	233.38	1	90.35	10.72	0.0015
Time	2.07	1	86.76	0.10	0.7584
Group X Time	0.32	1	86.74	0.01	0.9043
Baseline Peer Relationship T-score	225.14	1	91.58	10.34	0.0018
Parental Relationship T-score					
Group	424.86	1	93.78	12.24	0.0007
Time	3.34	1	91.83	0.10	0.7571
Group X Time	2.17	1	91.83	0.06	0.8030
Baseline Parental Relationship T-score	1101.69	1	93.36	31.73	0.0000
Social Stress T-score					
Group	295.65	1	90.21	5.27	0.0241
Time	385.88	1	89.88	6.87	0.0103
Group X Time	418.31	1	89.89	7.45	0.0076
Baseline Social Stress T-score	2229.20	1	89.87	39.70	0.0000

**Table 5:** Estimated means from mixed models

Group	Mean	s.e	Lower	Upper
Peer Relationship T-Score				
Control	51.80	1.40	49.10	54.60
Impact VR	58.10	1.30	55.50	60.70
	Mean	s.e	*t*	*p*
Difference	− 6.25	1.91	− 3.28	0.0015
Parental Relationship T-score				
Control	38.60	1.61	35.40	41.80
Impact VR	46.20	1.46	43.30	49.10
	Mean	s.e	*t*	*p*
Difference	− 7.61	2.18	− 3.50	0.0007
Social Stress Post-Intervention				
Control	53.00	1.40	50.20	55.80
Impact VR	52.40	1.33	49.80	55.00
	Mean	s.e	*t*	*p*
Difference	0.56	1.93	0.29	0.7705
Social Stress 3-Month Follow-Up				
Control	53.10	1.40	50.30	55.90
Impact VR	46.50	1.33	43.80	49.10
	Mean	s.e	*t*	*p*
Difference	6.66	1.93	3.45	0.0007

## Discussion

This RCT evaluated the efficacy of Impact VR, an immersive, youth-informed intervention designed to improve socioemotional functioning among adolescents with CD. Participants assigned to Impact VR showed significantly greater improvements than those in the active control group in emotion recognition, including total accuracy and specific recognition of sad, angry, and fearful expressions. Youth in the Impact VR condition also demonstrated improved peer relationships and relationships with parents, as well as reductions in social stress at follow-up, compared to those in the control group. These findings provide support for Impact VR as a promising tool to target core socioemotional processes associated with CD in adolescence.

These findings position Impact VR within a broader landscape of emerging approaches to strengthen socioemotional functioning among youth with conduct problems. By simultaneously addressing individual-level skills such as emotion recognition, relational processes involving peers and parents, and contextual barriers to engagement such as stigma and limited access (see Thomson et al., 2025a), Impact VR represents an innovative and mechanistically grounded intervention model. Impact VR represents the next generation approach for delivering socioemotional skill building in a brief, technology-based, and scalable format. Because the program requires minimal resources to deploy, it can be feasibly integrated alongside existing preventive and treatment efforts without additional staffing or training. In this way, Impact VR has the potential to complement multicomponent approaches by extending its reach and providing a developmentally engaging tool that can be delivered in schools, clinics, and community settings. Because sessions are self-guided and require minimal staffing, Impact VR may reduce personnel burden while increasing access, an important consideration given chronic resource shortages in youth behavioral health systems.

From a developmental perspective, the present study findings are consistent with theories highlighting the role of early social-cognitive disruptions in the emergence and persistence of antisocial behavior. The intervention was explicitly designed around SIP theory, which posits that aggression and relational difficulties are partly driven by biased or inaccurate interpretations of others’ emotions and intentions (Crick & Dodge, 1994). Youth with CD often show deficits in interpreting emotional cues (Martin‐Key et al., 2018), particularly distress-related emotions (Fairchild et al., 2009; Kleine Deters et al., 2020). These impairments can contribute to hostile attribution biases, poor conflict resolution, and challenges in forming or maintaining positive peer and caregiver relationships (Dodge et al., 1995; Lansford et al., 2006). By directly targeting emotion recognition through immersive, developmentally relevant learning experiences, Impact VR may recalibrate these encoding processes and reduce misunderstandings while improving social functioning.

This study also found that youth who participated in Impact VR showed significant improvements in interpersonal functioning and parental relationships. The changes observed in social information encoding and interpretation may also strengthen the relationships held by youth with CD, potentially reducing biases and/or improving the accuracy of decoding others' emotions. These perceptual shifts in social cue encoding and interpretation may support more adaptive relationship patterns with peers and parents. Building emotion recognition skills increases youths’ capacity for empathy (Benitez-Lopez & Ramos-Loyo, 2022; Dadds et al., 2012), which is critical for interpersonal relationships with peers (Fink & Rosnay, 2023) and for secure attachment to both peers and caregivers (Xu et al., 2022). Thus, youth can build stronger interpersonal connections by improving their emotion recognition ability. This is particularly salient as associations with “nondeviant” peers can function as a protective factor for later antisocial behaviors (Vitaro et al., 1999). Similarly, strong parent–child relationships, hallmarked by communication, trust, and empathy, have been found to be a protective factor for antisocial and aggressive behavior in adolescents (Guilamo-Ramos et al., 2006; Van der Graaff et al., 2012). Thus, the skills gained from Impact VR aid in the ability to build upon and strengthen interpersonal relationships through empathetic understanding and accurate encoding skills, which in turn may further help to reduce the severity of conduct problems.

The results also show that youth demonstrated a significant reduction in social stress at the 3-month follow-up, but not immediately post-intervention. Prior literature emphasizes the importance of emotion recognition skills and appropriate responses to social-emotional stimuli for social competence (Trentacosta & Fine, 2010), social adjustment (Leppänen & Hietanen, 2001), and empathy, commitment, and self-control in social settings (Benitez-Lopez & Ramos-Loyo, 2022). By improving upon this social functioning skillset, it follows that youth feel more confident navigating various social settings, thereby reducing their overall social stress (e.g., worries about being excluded). Furthermore, research has demonstrated that accurate social cue encoding and interpretation improves self-esteem (Wells et al., 2020), which further helps to mitigate social stress (Shang et al., 2025). The observed reduction in social stress at 3-month follow-up, but not immediately post-intervention, suggests that youth may require time to integrate these skills into real-world contexts. This lag is consistent with generalization models of learning, which emphasize that skill transfer often depends on opportunities to apply newly learned behaviors across varied social settings (Stokes & Baer, 1977). The immersive nature of the VR platform may have facilitated the encoding and rehearsal of these skills. However, sustained improvements in social stress likely required continued engagement with peers and caregivers outside the intervention itself. This is encouraging that improvements can be seen beyond the implementation of such a brief intervention.

These results contribute to a growing body of literature on emotion recognition interventions for youth with externalizing behaviors. Prior studies using static or slideshow-based training methods have shown limited or short-term effects (e.g., Muñoz Centifanti et al., 2021). In contrast, Impact VR incorporates dynamic scenarios, real-time feedback, and narrative-driven engagement; these components are hypothesized to increase retention and relevance, especially for youth with attention or motivation difficulties. The positive outcomes observed in this study suggest that immersive delivery may enhance both the intensity and ecological validity of emotion recognition training. Furthermore, it has been shown that gamification enhances learning outcomes through increasing motivation, engagement, and retention (Jaramillo-Mediavilla et al., 2024; M. Li et al., 2023a, b). Thus, Impact VR offers a novel experience for youth to engage in emotion recognition training and build fundamental skills for success in social-emotional development.

A key strength of this trial is the use of an active control group, which allows for greater confidence that the observed effects were attributable to the specific content and delivery of Impact VR, rather than nonspecific factors such as attention or novelty. In addition, rather than administering this intervention solely in a controlled laboratory setting, this trial utilized the youths’ home environment. By delivering the intervention to youth at home, this trial was able to replicate one of the key conditions in which Impact VR would likely be implemented following broader distribution. Thus, this allows for a greater generalization of these findings across various settings and suggests that the results are indicative of the intervention’s real-world effectiveness. Additional strengths include the randomized controlled design, blinded baseline assessments, and use of both youth reports and neurocognitive assessments, which increase internal validity and reduce the risk of bias. Finally, the sample was racially and ethnically diverse and recruited from a public healthcare network, supporting the potential generalizability of the findings to real-world clinical populations rather than focusing exclusively on youth in detention.

Several limitations should be noted. First, the present study used the ER-40 as the outcome measure for emotion recognition accuracy. Although this is a widely used assessment, it has two weaknesses in this context. Impact VR trains recognition across a range of age groups, including adults, adolescents, and children, but the ER-40 presents only adult faces. This narrows the scope of improvements that can be captured. In addition, the maximum correct score for each emotion on the ER-40 is eight, and youth in both groups were already performing near ceiling on happy faces (*M* = 7.73 out of 8). This left little room for observable improvement in that category. By contrast, recognition of fear, sadness, and anger, domains consistently identified as areas of deficit for youth with conduct problems, showed more room for growth and demonstrated significant intervention-related gains. This pattern highlights the importance of selecting tasks with sufficient headroom for change in prevention trials. Future studies may benefit from incorporating peer-aged or more ambiguous expressions to increase sensitivity while retaining continuity with widely used measures like the ER-40.

Second, this study was not designed to evaluate long-term outcomes, and it remains unknown whether the gains observed here persist beyond the three-month follow-up. Future research should assess whether improvements in emotion recognition and interpersonal functioning translate into longer-term behavioral change or serve as protective factors against the progression of conduct problems. Although Impact VR improved socioemotional functioning over time, it was compared with a brief, one-time active control rather than a more intensive evidence-based treatment such as CBT. Because participants in the Impact VR group completed four sessions compared to one in the control condition, it remains unclear how Impact VR would perform relative to established treatments, and whether the observed effects reflect unique intervention mechanisms or simply greater exposure and engagement. This design choice reflected the need for a feasible and cost-efficient comparator in an early-phase trial, and one that has demonstrated effectiveness in prior research across multiple contexts (Dadds et al., 2006; Muñoz Centifanti et al., 2021; Preis et al., 2025); however, future work should incorporate dose-matched active controls or treatment-as-usual conditions to more clearly isolate the mechanisms responsible for change. Lastly, the study was not designed to assess the economic efficiency of the intervention. Future research should directly compare Impact VR with established evidence-based treatments to evaluate relative cost-effectiveness. Such analyses would help determine whether self-directed technology-based approaches can achieve comparable outcomes with fewer resources or offer advantages in accessibility, dissemination, and scalability, particularly in under-resourced or high-need settings. Although sex and age were examined as potential moderators, neither meaningfully influenced intervention outcomes. These findings suggest that Impact VR produced similar benefits across boys and girls and across the adolescent age range.

In conclusion, Impact VR represents a novel and developmentally sensitive approach to improving socioemotional functioning in males and females with conduct disorder. By targeting social-emotional learning through immersive virtual experiences, the program addresses key mechanisms that contribute to interpersonal difficulties and social stress in this population. Findings from this study indicate that even brief interventions can produce meaningful improvements in core socioemotional domains. As such, Impact VR may offer a scalable, engaging, and culturally relevant addition to the prevention science toolbox for supporting youth with conduct problems.

## Data Availability

The study dataset is not publicly available due to privacy restrictions, but may be obtained from the corresponding author upon reasonable request and completion of a data use agreement.

## Footnotes

1 Sex and age were examined as potential moderators in separate models that included the three-way and two-way interaction terms (sex × group × time, sex × group, sex × time; age × group × time, age × group, age × time). No sex interactions were statistically significant for all outcomes. A significant age × group × time interaction was detected for the sad subscale on the ER40 (*p* = .0239), but follow-up tests at the mean and ± 1 SD of age were not statistically significant after Bonferroni correction. These results indicate that Impact VR improved outcomes regardless of sex or age.

## Declarations

### Preregistration

This trial was preregistered on ClinicalTrials.gov (Identifier: NCT06301516) prior to participant enrollment.

## References

[CR1] Adolphs, R. (2006). Perception and emotion: How we recognize facial expressions. *Current Directions in Psychological Science : A Journal of the American Psychological Society,**15*(5), 222–226. 10.1111/j.1467-8721.2006.00440.x

[CR2] American Psychiatric Association. (2022). *Diagnostic and statistical manual of mental disorders: DSM-5-TR*. American Psychiatric Association. (DSM-5-TR).10.1176/appi.books.9780890425787

[CR3] Arche XR. (2024). Impact VR (Version V1) [Computer software]. Arche XR. www.ArcheXR.com

[CR4] Benitez-Lopez, Y., & Ramos-Loyo, J. (2022). Improved ability in emotional recognition and social skills after emotional recognition training in children. *International Journal of Psychological Studies,**14*(3), Article 1. 10.5539/ijps.v14n3p137799376

[CR5] Blair, R. J. R. (2013). The neurobiology of psychopathic traits in youths. *Nature Reviews Neuroscience,**14*(11), 786–799. 10.1038/nrn3577 24105343 10.1038/nrn3577 PMC4418507

[CR6] Blondell, V. J., & Thomson, N. D. (2025). Virtual Reality as an Innovative Tool for Youth Mental Health. *Encyclopedia, 5*(4), 215.

[CR7] Cohen, J. (2013). *Statistical power analysis for the behavioral sciences* (Rev. ed). Academic Press.

[CR8] Crick, N. R., & Dodge, K. A. (1994). A review and reformulation of social information-processing mechanisms in children’s social adjustment. *Psychological Bulletin,**115*(1), 74–101. 10.1037/0033-2909.115.1.74

[CR9] Dadds, M. R., Cauchi, A. J., Wimalaweera, S., Hawes, D. J., & Brennan, J. (2012). Outcomes, moderators, and mediators of empathic-emotion recognition training for complex conduct problems in childhood. *Psychiatry Research,**199*(3), 201–207. 10.1016/j.psychres.2012.04.033 22703720 10.1016/j.psychres.2012.04.033

[CR10] Dadds, M. R., Perry, Y., Hawes, D. J., Merz, S., Riddell, A. C., Haines, D. J., ... & Abeygunawardane, A. I. (2006). Attention to the eyes and fear-recognition deficits in child psychopathy. *The British Journal of Psychiatry, 189*(3), 280-281. 10.1192/bjp.bp.105.018150 16946366

[CR11] Dadds, M. R., Kimonis, E. R., Schollar-Root, O., Moul, C., & Hawes, D. J. (2018). Are impairments in emotion recognition a core feature of callous–unemotional traits? Testing the primary versus secondary variants model in children. *Development and Psychopathology,**30*(1), 67–77. 10.1017/S0954579417000475 28420457 10.1017/S0954579417000475

[CR12] De Brito, S. A., Forth, A. E., Baskin-Sommers, A. R., Brazil, I. A., Kimonis, E. R., Pardini, D., Frick, P. J., Blair, R. J. R., & Viding, E. (2021). Psychopathy. *Nature Reviews. Disease Primers, 7*(1), 49. 10.1038/s41572-021-00282-1 10.1038/s41572-021-00282-1 34238935

[CR13] Díaz-Vázquez, B., López-Romero, L., & Romero, E. (2024). Emotion recognition deficits in children and adolescents with psychopathic traits: A systematic review. *Clinical Child and Family Psychology Review,**27*(1), 165–219. 10.1007/s10567-023-00466-z 38240937 10.1007/s10567-023-00466-z PMC10920463

[CR14] Dodge, K. A. (1993). Social-cognitive mechanisms in the development of conduct disorder and depression. *Annual Review of Psychology,**44*(1), 559–584. 10.1146/annurev.ps.44.020193.003015 8434896 10.1146/annurev.ps.44.020193.003015

[CR15] Dodge, K. A., & Pettit, G. S. (2003). A biopsychosocial model of the development of chronic conduct problems in adolescence. *Developmental Psychology,**39*(2), 349–371. 10.1037/0012-1649.39.2.349 12661890 10.1037//0012-1649.39.2.349 PMC2755613

[CR16] Dodge, K. A., Pettit, G. S., Bates, J. E., & Valente, E. (1995). Social information-processing patterns partially mediate the effect of early physical abuse on later conduct problems. *Journal of Abnormal Psychology,**104*(4), 632–643. 10.1037/0021-843X.104.4.632 8530766 10.1037//0021-843x.104.4.632

[CR17] Efird, J. (2011). Blocked randomization with randomly selected block sizes. *International Journal of Environmental Research and Public Health,**8*(1), 15–20. 10.3390/ijerph8010015 21318011 10.3390/ijerph8010015 PMC3037057

[CR18] Erskine, H. E., Ferrari, A. J., Polanczyk, G. V., Moffitt, T. E., Murray, C. J. L., Vos, T., Whiteford, H. A., & Scott, J. G. (2014). The global burden of conduct disorder and attention-deficit/hyperactivity disorder in 2010. *Journal of Child Psychology and Psychiatry,**55*(4), 328–336. 10.1111/jcpp.12186 24447211 10.1111/jcpp.12186

[CR19] Fairchild, G., Hawes, D., Frick, P., Copeland, W., Odgers, C., Franke, B., Freitag, C., & De Brito, S. (2019). *Conduct disorder*. 10.1038/s41572-019-0095-y 31249310

[CR20] Fairchild, G., Van Goozen, S. H. M., Calder, A. J., Stollery, S. J., & Goodyer, I. M. (2009). Deficits in facial expression recognition in male adolescents with early-onset or adolescence-onset conduct disorder. *Journal of Child Psychology and Psychiatry,**50*(5), 627–636. 10.1111/j.1469-7610.2008.02020.x 19432683 10.1111/j.1469-7610.2008.02020.x PMC2737612

[CR21] Farashi, S., Bashirian, S., Jenabi, E., & Razjouyan, K. (2024). Effectiveness of virtual reality and computerized training programs for enhancing emotion recognition in people with autism spectrum disorder: A systematic review and meta-analysis. *International Journal of Developmental Disabilities,**70*(1), 110. 10.1080/20473869.2022.2063656 38456137 10.1080/20473869.2022.2063656 PMC10916911

[CR22] Ferrari, A., Santomauro, D., Herrera, A., Shadid, J., Ashbaugh, C., Erskine, H., Charlson, F., Degenhardt, L., Scott, J., & McGrath, J. (2022). Global, regional, and national burden of 12 mental disorders in 204 countries and territories, 1990–2019: A systematic analysis for the Global Burden of Disease Study 2019. *Lancet Psychiatry,**9*(2), 137–150. 10.1016/S2215-0366(21)00395-3 35026139 10.1016/S2215-0366(21)00395-3 PMC8776563

[CR23] Fink, E., & Rosnay, M. (2023). Examining links between affective empathy, cognitive empathy, and peer relationships at the transition to school. *Social Development (Oxford, England),**32*(4), 1208–1226. 10.1111/sode.12685

[CR24] Frick, P. J., & Viding, E. (2009). Antisocial behavior from a developmental psychopathology perspective. *Development and Psychopathology,**21*(4), 1111–1131. 10.1017/S0954579409990071 19825260 10.1017/S0954579409990071

[CR25] Fleming, G. E., Neo, B., Briggs, N. E., Kaouar, S., Frick, P. J., & Kimonis, E. R. (2022). Parent training adapted to the needs of children with callous–unemotional traits: A randomized controlled trial. *Behavior therapy, 53*(6), 1265-1281. 10.1016/j.beth.2022.07.001 36229121

[CR26] Guilamo-Ramos, V., Jaccard, J., Dittus, P., & Bouris, A. M. (2006). Parental expertise, trustworthiness, and accessibility: Parent-adolescent communication and adolescent risk behavior. *Journal of Marriage and Family,**68*(5), 1229–1246. 10.1111/j.1741-3737.2006.00325.x

[CR27] Herba, C., & Phillips, M. (2004). Annotation: Development of facial expression recognition from childhood to adolescence: Behavioural and neurological perspectives. *Journal of Child Psychology and Psychiatry,**45*(7), 1185–1198. 10.1111/j.1469-7610.2004.00316.x 15335339 10.1111/j.1469-7610.2004.00316.x

[CR28] Hubble, K., Bowen, K. L., Moore, S. C., & van Goozen, S. H. M. (2015). Improving negative emotion recognition in young offenders reduces subsequent crime. *PLoS ONE,**10*(6), Article e0132035. 10.1371/journal.pone.0132035 26121148 10.1371/journal.pone.0132035 PMC4486167

[CR29] Hunnikin, L. M., Wells, A. E., Ash, D. P., & van Goozen, S. H. M. (2022). Can facial emotion recognition be rapidly improved in children with disruptive behavior? A targeted and preventative early intervention study. *Development and Psychopathology,**34*(1), 85–93. 10.1017/S0954579420001091 33432899 10.1017/S0954579420001091

[CR30] Izard, C., Fine, S., Schultz, D., Mostow, A., Ackerman, B., & Youngstrom, E. (2001). Emotion knowledge as a predictor of social behavior and academic competence in children at risk. *Psychological Science,**12*(1), 18–23. 10.1111/1467-9280.00304 11294223 10.1111/1467-9280.00304

[CR31] Jaramillo-Mediavilla, L., Basantes-Andrade, A., Cabezas-González, M., & Casillas-Martín, S. (2024). Impact of gamification on motivation and academic performance: A systematic review. *Education Sciences,**14*(6), Article 639. 10.3390/educsci14060639

[CR32] Kevorkian, S. S., & Thomson, N. D. (2024). Virtual reality: A promising new strategy for hospital-based violence interventions for Spanish-speaking patients and in latin America.* Panamerican Journal of Trauma, Critical Care & Emergency Surgery, 13*, 10-1.

[CR33] Kimonis, E. R., Fleming, G., Briggs, N., Brouwer-French, L., Frick, P. J., Hawes, D. J., ... & Dadds, M. (2019). Parent-child interaction therapy adapted for preschoolers with callous-unemotional traits: An open trial pilot study. *Journal of Clinical Child & Adolescent Psychology, 48*(sup1), S347-S361. 10.1080/15374416.2018.1479966 29979887

[CR34] Kleine Deters, R., Naaijen, J., Rosa, M., Aggensteiner, P. M., Banaschewski, T., Saam, M. C., Schulze, U. M. E., Sethi, A., Craig, M. C., Sagar-Ouriaghli, I., Santosh, P., Castro-Fornieles, J., Penzol, M. J., Arango, C., Werhahn, J. E., Brandeis, D., Franke, B., Glennon, J., Buitelaar, J. K., …, Dietrich, A. (2020). Executive functioning and emotion recognition in youth with oppositional defiant disorder and/or conduct disorder. *The World Journal of Biological Psychiatry*, *21*(7), 539–551. 10.1080/15622975.2020.1747114 10.1080/15622975.2020.1747114 32212964

[CR35] Koerting, J., Smith, E., Knowles, M. M., Latter, S., Elsey, H., McCann, D. C., Thompson, M., & Sonuga-Barke, E. J. (2013). Barriers to, and facilitators of, parenting programmes for childhood behaviour problems: A qualitative synthesis of studies of parents’ and professionals’ perceptions. *European Child & Adolescent Psychiatry,**22*(11), 653–670. 10.1007/s00787-013-0401-2 23564207 10.1007/s00787-013-0401-2 PMC3826057

[CR36] Kohler, C. G., Turner, T. H., Bilker, W. B., Brensinger, C. M., Siegel, S. J., Kanes, S. J., . . . Gur, R. C. (2003). Facial emotion recognition in schizophrenia: Intensity effects and error pattern. *American Journal of Psychiatry, 160*(10), 1768-1774. 10.1176/appi.ajp.160.10.1768 14514489

[CR37] Lansford, J. E., Malone, P. S., Stevens, K. I., Dodge, K. A., Bates, J. E., & Pettit, G. S. (2006). Developmental trajectories of externalizing and internalizing behaviors: Factors underlying resilience in physically abused children. *Development and Psychopathology,**18*(1), 35–55. 10.1017/S0954579406060032 16478551 10.1017/S0954579406060032 PMC2772062

[CR38] Leppänen, J. M., & Hietanen, J. K. (2001). Emotion recognition and social adjustment in school-aged girls and boys. *Scandinavian Journal of Psychology,**42*(5), 429–435. 10.1111/1467-9450.00255 11771812 10.1111/1467-9450.00255

[CR39] Li, F., Li, X., & Kou, H. (2023). Emotional recognition training enhances attention to emotional stimuli among male juvenile delinquents. *Psychology Research and Behavior Management,**16*, 575–586. 10.2147/PRBM.S403512 36883045 10.2147/PRBM.S403512 PMC9985883

[CR40] Li, M., Ma, S., & Shi, Y. (2023). Examining the effectiveness of gamification as a tool promoting teaching and learning in educational settings: A meta-analysis. *Frontiers in Psychology,**14*, Article 1253549. 10.3389/fpsyg.2023.1253549 37876838 10.3389/fpsyg.2023.1253549 PMC10591086

[CR41] Li, X., Kou, H., Bi, T., & Peng, Z. (2024). Deficits in emotional cognition among individuals with conduct disorder: Theoretical perspectives. *Frontiers in Psychiatry,**15*, Article 1507695. 10.3389/fpsyt.2024.1507695 39720439 10.3389/fpsyt.2024.1507695 PMC11666480

[CR42] Mancuso, V., Bruni, F., Stramba-Badiale, C., Riva, G., Cipresso, P., & Pedroli, E. (2023). How do emotions elicited in virtual reality affect our memory? A systematic review. *Computers in Human Behavior,**146*, Article 107812. 10.1016/j.chb.2023.107812

[CR43] Martin-Key, N. A., Graf, E. W., Adams, W. J., & Fairchild, G. (2018). Facial emotion recognition and eye movement behaviour in conduct disorder. *Journal of Child Psychology and Psychiatry,**59*(3), 247–257. 10.1111/jcpp.12795 28881001 10.1111/jcpp.12795

[CR44] Mondloch, C. J., Geldart, S., Maurer, D., & Grand, R. L. (2003). Developmental changes in face processing skills. *Journal of Experimental Child Psychology,**86*(1), 67–84. 10.1016/S0022-0965(03)00102-4 12943617 10.1016/s0022-0965(03)00102-4

[CR45] Montirosso, R., Peverelli, M., Frigerio, E., Crespi, M., & Borgatti, R. (2010). The development of dynamic facial expression recognition at different intensities in 4- to 18-year-olds. *Social Development (Oxford, England),**19*(1), 71–92. 10.1111/j.1467-9507.2008.00527.x

[CR46] Muñoz Centifanti, L. C., Stickle, T. R., Thomas, J., Falcón, A., Thomson, N. D., & Gamer, M. (2021). Reflexive gaze shifts and fear recognition deficits in children with callous-unemotional traits and impulsivity/conduct problems. *Brain Sciences,**11*(10), Article 1342. 10.3390/brainsci11101342 34679406 10.3390/brainsci11101342 PMC8533769

[CR47] Parsons, S. (2016). Authenticity in virtual reality for assessment and intervention in autism: A conceptual review. *Educational Research Review,**19*, 138–157. 10.1016/j.edurev.2016.08.001

[CR48] Preis, M. A., Schlegel, K., Rehbein, S., Lorenz, K., & Brockmeyer, T. (2025). Training emotion recognition in depression—An experimental study. *British Journal of Clinical Psychology*. 10.1111/bjc.12540 PMC12334984 40098580

[CR49] R Core Team. (2025). *R: A language and environment for statistical computing* [Computer software]. R Foundation for Statistical Computing. https://www.R-project.org/. Accessed August 2025.

[CR50] Reynolds, C. R., & Kamphaus, R. W. (2015). *Behavior assessment system for children—Third Edition (BASC-3) manual*. Pearson.

[CR51] Reed, Z. E., Suddell, S., Eastwood, A., Thomas, L., Dwyer, I., Penton-Voak, I. S., ... & Attwood, A. S. (2023). Assessing the effectiveness of online emotion recognition training in healthy volunteers. *Royal Society Open Science, 10*(9), 230372. 10.1098/rsos.230372 PMC10523077 37771966

[CR52] Sebastian, C. L., Stafford, J., McCrory, E. J., Sethi, A., De Brito, S. A., Lockwood, P. L., & Viding, E. (2021). Modulation of amygdala response by cognitive conflict in adolescents with conduct problems and varying levels of CU traits. *Journal of Abnormal Child Psychology,**49*(8), 1043–1054. 10.1007/s10802-021-00787-z 10.1007/s10802-021-00787-z PMC8222043 33728508

[CR53] Sethi, S. S., & Jain, K. (2024). AI technologies for social emotional learning: Recent research and future directions. *Journal of Research in Innovative Teaching & Learning,**17*(2), 213–225. 10.1108/JRIT-03-2024-0073

[CR54] Shang, A., Feng, L., Yan, G., & Sun, L. (2025). The relationship between self-esteem and social avoidance among university students: Chain mediating effects of resilience and social distress. *BMC Psychology,**13*(1), 116–211. 10.1186/s40359-025-02444-2 39934905 10.1186/s40359-025-02444-2 PMC11816749

[CR55] Stokes, T. F., & Baer, D. M. (1977). An implicit technology of generalization. *Journal of Applied Behavior Analysis,**10*(2), 349–367. 10.1901/jaba.1977.10-349 16795561 10.1901/jaba.1977.10-349 PMC1311194

[CR56] Thomson, N. D., Centifanti, L. C. M., & Lemerise, E. A. (2017). Emotion regulation and conduct disorder: The role of callous-unemotional traits. In C. A. Essau, S. Leblanc, & T. H. Ollendick (Eds.), *Emotion regulation and psychopathology in children and adolescents* (pp. 129–153). Oxford University Press. 10.1093/med:psych/9780198765844.003.0007

[CR57] Thomson, N. D., Kevorkian, S. S., Hazlett, L., Perera, R., & Vrana, S. (2025). A new treatment approach to conduct disorder and callous-unemotional traits: An assessment of the acceptability, appropriateness, and feasibility of Impact VR. *Frontiers in Psychiatry,**16*, Article 1484938. 10.3389/fpsyt.2025.1484938 40375881 10.3389/fpsyt.2025.1484938 PMC12078265

[CR58] Thomson, N. D., Perera, R. A., Kevorkian, S. S., Hazlett, L., & Vrana, S. (2025). Impact VR: A socioemotional intervention for reducing CU traits, conduct problems, and aggression in youth with conduct disorder. *Research on Child and Adolescent Psychopathology*. 10.1007/s10802-025-01373-3 41055883 10.1007/s10802-025-01373-3 PMC12718285

[CR59] Trentacosta, C. J., & Fine, S. E. (2010). Emotion knowledge, social competence, and behavior problems in childhood and adolescence: A meta-analytic review. *Social Development,**19*(1), 1–29. 10.1111/j.1467-9507.2009.00543.x 21072259 10.1111/j.1467-9507.2009.00543.x PMC2975582

[CR60] Van der Graaff, J., Branje, S., De Wied, M., & Meeus, W. (2012). The moderating role of empathy in the association between parental support and adolescent aggressive and delinquent behavior. *Aggressive Behavior,**38*(5), 368–377. 10.1002/ab.21435 22898874 10.1002/ab.21435

[CR61] Vié, C., Govindin-Ramassamy, K., Thellier, D., Labrosse, D., & Montagni, I. (2024). Effectiveness of digital games promoting young people’s mental health: A review of reviews. *Digital Health,**10*, 20552076231220816. 10.1177/20552076231220814 38323239 10.1177/20552076231220814 PMC10845979

[CR62] Vitaro, F., Brendgen, M., Pagani, L., Tremblay, R. E., & McDuff, P. (1999). Disruptive behavior, peer association, and conduct disorder: Testing the developmental links through early intervention. *Development and Psychopathology,**11*(2), 287–304. 16506535 10.1017/s0954579499002060

[CR63] Wells, A. E., Hunnikin, L. M., Ash, D. P., & van Goozen, S. H. M. (2020). Low self-esteem and impairments in emotion recognition predict behavioural problems in children. *Journal of Psychopathology and Behavioral Assessment,**42*(4), 693–701. 10.1007/s10862-020-09814-7

[CR64] Wells, A. E., Hunnikin, L. M., Ash, D. P., & van Goozen, S. H. M. (2021). Improving emotion recognition is associated with subsequent mental health and well-being in children with severe behavioural problems. *European Child & Adolescent Psychiatry,**30*(11), 1769–1777. 10.1007/s00787-020-01652-y 32997168 10.1007/s00787-020-01652-y PMC8558267

[CR65] Woicik, K., Geraets, C. N. W., Klein Tuente, S., Masthoff, E., & Veling, W. (2023). Virtual reality aggression prevention treatment in a Dutch prison-based population: A pilot study. *Frontiers in Psychology,**14*, Article 1235808. 10.3389/fpsyg.2023.1235808 38034305 10.3389/fpsyg.2023.1235808 PMC10683795

[CR66] Xu, X., Liu, Z., Gong, S., & Wu, Y. (2022). The relationship between empathy and attachment in children and adolescents: Three-level meta-analyses. *International Journal of Environmental Research and Public Health,**19*(3), Article 1391. 10.3390/ijerph19031391 35162410 10.3390/ijerph19031391 PMC8835466

[CR67] Zhang, F., Zhang, Y., Li, G., & Luo, H. (2024). Using virtual reality interventions to promote social and emotional learning for children and adolescents: A systematic review and meta-analysis. *Children (Basel),**11*(1), 41. 10.3390/children11010041 10.3390/children11010041 PMC10813885 38255355

